# The diagnostic performance of a noninvasive urine-based methylation biomarkers Vimentin/POU4F2 to detect bladder carcinoma

**DOI:** 10.1186/s12885-025-14795-5

**Published:** 2025-09-30

**Authors:** Jialong Zhang, Xiaowei Cheng, Cong Huang, Yuchen Xu, Jun He, Hanjiang Xu, Sheng Tai, Yujun Wei

**Affiliations:** 1 Department of Urology, The First Affiliated Hospital of Anhui Medical University, Hefei, China; 2 Department of Respiratory and Critical Care Medicine, Panzhihua Municipal Central Hospital, Panzhihua, China; 3 Anhui Anlong Gene Technology Co, Ltd, Anhui China

**Keywords:** Bladder urothelial carcinoma, Urine, Methylation biomarkers, Vimentin, POU4F2

## Abstract

**Background:**

Bladder carcinoma (BC) is a malignant tumor that originates from the epithelial cells of the urinary system. Currently, the main diagnostic methods suffer from disadvantages including being invasive, having high costs, and low sensitivity and specificity. Therefore, there is an urgent need to develop a non-invasive, high-performance method for diagnosing BC.

**Materials and methods:**

We developed a urine DNA detection panel utilizing two methylation biomarkers for the diagnosis of BC. A total of 467 urine samples were collected from the First Affiliated Hospital of Anhui Medical University for DNA methylation analysis. The methylation levels of Vimentin and POU class 4 homeobox 2 gene (POU4F2) were analyzed in a training set of 306 urine samples (92 cases and 214 controls), and in an independent validation set of 161 urine samples (59 cases and 102 controls) using Real-Time PCR (RT-PCR).

**Results:**

The Vimentin/POU4F2 combined methylation panel achieved an AUC of 0.935 (95% CI: 0.889–0.981), with sensitivity, specificity, and accuracy of 86.44% (95% CI: 0.772–0.957), 96.08% (95% CI: 0.923–0.998), and 92.55% (95% CI: 0.886–0.965) for diagnosing BC. Notably, for patients with stage I and low-grade BC, the sensitivity was 90.00% for both. Additionally, it demonstrated specificities of 96.30% and 95.83% for patients with other urinary diseases and malignancies in other systems.

**Conclusions:**

Our study provides evidence that the two-gene methylation panel based on urine DNA detection demonstrates strong performance in diagnosing BC with high sensitivity and accuracy, offering a promising strategy for early screening and adjunctive diagnosis of BC.

**Supplementary Information:**

The online version contains supplementary material available at 10.1186/s12885-025-14795-5.

## Introduction

Urothelial carcinomas (UCs) are malignant tumors that occur in the urothelium, and are among the most common malignant tumors in the urogenital system [1, 2]. They can occur in anywhere covered by the urothelium, including the renal pelvis, ureter, bladder, urethra, and urachus. Among them, BC accounts for the vast majority (90%−95%) of all UCs [3, 4]. According to the latest data from the National Cancer Center of China (NCC), the incidence and mortality rates of BC in 2022 were 9.3 and 4.1 per 100,000, respectively. Therefore, early detection and treatment of BC are crucial.

Currently, BC screening methods primarily include cystourethroscopy, ultrasound, urine cytology, and computed tomography/computed tomography urography (CT/CTU). Although cystourethroscopy is the gold standard for diagnosing BC [5], it is costly and invasive, with potential complications such as swelling, bleeding, infection, and urosepsis, and carries the risk of further complications such as urosepsis [6]. Ultrasound examination is non-invasive, economical, radiation-free, and easy to perform, but its accuracy in diagnosing BC is closely related to the degree of bladder filling, the size, shape, and location of the tumor, and is also significantly affected by obesity or gas in the intestinal cavity [7]. CT/CTU is the most valuable imaging examination for diagnosing BC, with a sensitivity of 67%. However, the use of radiation and contrast agents may affect patients’ health; some patients with renal insufficiency may not be able to tolerate this examination [8]. Regarding other auxiliary methods, such as conventional urine cytology, studies have shown that its sensitivity for high-grade BC is 84%, but only 16% for low-grade BC [9]. More importantly, cytology is less sensitive for upper tract urothelial carcinoma (UTUC) than for BC [10]. Therefore, it is imperative to develop a simple, cost-effective, reliable, and non-invasive method to expedite the diagnosis of BC.

In recent years, an increasing number of studies have found that the epigenetic molecular regulatory network plays a significant role in tumor development and progression [11]. DNA methylation, a common epigenetic modification, is typically associated with gene expression silencing and is well-characterized [12]. Hypermethylation of tumor suppressor gene promoters is an early event in many cancers and can be detected in the early stages [13]. Several studies indicate that bodily fluids, including urine, gastric juice, plasma/serum, and sputum [14, 15], can be used for non-invasive cancer detection via DNA hypermethylation. During the occurrence and development of UCs, urothelial tumors persist in the urine environment for extended periods. A large number of tumor cells and DNA fragments are easily shed into the urine and subsequently excreted [16]. Therefore, detecting specific gene methylation changes in exfoliated cells from urine samples can aid in the early diagnosis of clinically suspected BC patients. However, most published DNA methylation-based panels improve sensitivity at the expense of reduced specificity. For example, a previous study demonstrated that TWIST1/Vimentin promoter methylation in urine samples for differentiating BC from benign diseases and healthy controls achieved sensitivities of 78% and specificities of 83%, respectively [17]. Another study suggested that a panel combining the methylation biomarkers HOXA9, PCDH17, POU4F2, and ONECUT2 yielded an area under the receiver operating characteristic curve of 0.871 with a sensitivity of 90.5% and specificity of 73.2% [18]. The specificities in the above studies were lower compared to those of fluorescence in situ hybridization (FISH) and cytology [19, 20]. Therefore, there is an urgent need to screen for a urine methylation biomarkers panel with high sensitivity and specificity to improve the efficiency of the early diagnosis of bladder cancer.

The POU class 4 homeobox 2 gene (POU4F2), also known as BRN3B, belongs to the POU-domain transcription factor family and encodes a protein that plays a crucial role in regulating gene expression [21]. Studies have shown that hypermethylation of the POU4F2 promoter region is frequently observed and may serve as a promising biomarker for BC. A study involving 312 participants found that combining POU4F2 and PCDH17 methylation assays achieved a sensitivity of 90.00% and a specificity of 93.96% in detecting BC [18], highlighting the potential of POU4F2 methylation as a biomarker for the non-invasive detection of UC. Vimentin is a type III intermediate filament protein predominantly expressed in mesenchymal cells [22]. It plays a vital role in maintaining cellular integrity, promoting migration, and mediating signaling. In cancer, Vimentin is closely associated with epithelial-to-mesenchymal transition (EMT), a process that enhances tumor invasiveness and metastasis [23, 24]. One study demonstrated that VIM promoter methylation, along with biomarkers such as GDF15 and TMEFF2, accurately identified BC in both tissue and urine samples. The assay achieved 91% sensitivity and 100% specificity in urine samples [25]. Another study reported that a methylation panel comprising GDF15, HSPA2T, TMEFF2, and VIM achieved 94% sensitivity, 100% specificity, and an area under the curve (AUC) of 0.975 in urine samples [26]. These findings suggest that VIM gene methylation is a promising biomarker for improving early detection of BC. Although multi-methylation marker combinations have become a mainstream strategy for improving the non-invasive diagnostic performance of BC [27, 28], the specific combination of Vimentin and POU4F2 has not been previously reported. This study is the first to investigate the diagnostic utility of this combination. The design concept of this combination aims to integrate Vimentin’s high detection ability for invasive tumors and POU4F2’s high sensitivity for early tumors, with the goal of achieving more comprehensive and robust detection across different stages/grades of BC.

In this study, we aimed to develop a non-invasive liquid biopsy assay capable of distinguishing BC from other urinary diseases using a combined Vimentin/POU4F2 methylation panel. The methylation panel demonstrated high sensitivity, specificity, and accuracy in detecting BC, particularly in stage I patients, indicating its strong potential for early screening and diagnosis. However, its diagnostic performance requires further testing with larger, independent clinical cohorts.

## Materials and methods

### Sample size for adequate sensitivity/specificity

The formula for estimating the required sample size at a (1-α) % confidence level is derived as follows: P represents the pre-determined value of sensitivity (or specificity); Z_1-α/2_ is the z-value from the standard normal distribution corresponding to a left-tail probability of (1-α/2); and ∆ is one half the desired width of the confidence interval. This study was designed to evaluate a sensitivity and specificity of 90% with 95% confidence and a maximum allowable margin of error of 0.05. Based on this criterion, at least 139 cancer samples and 73 healthy/benign samples were required in the dataset.

$$\mathrm n=\;\frac{\left({\mathrm Z}_{1-\mathrm\alpha/2}\right)^2\mathrm P\left(1-\mathrm P\right)}{\triangle^2}$$

### Primer and probe design

Primer and probe design: Primers and probes for Vimentin and POU4F2 were designed based on the DNA sequences after bisulfite conversion, targeting the dense CpG island regions in the gene promoter areas (Vimentin: Chr10 + 17,270,244–17272636; POU4F2: Chr7: + 147,559,626–147,560,645) (Fig. S1 and S2). All primers were validated for product specificity using melting curve analysis and gel electrophoresis.

Primer and probe design principles: The probes spanned ≥ 3 CpG sites, with the 5’ end labeled with FAM/ROX and the 3’ end quenched with BHQ1/BHQ2. Primer length ranged from 18 to 22 bp, with a Tm value of 58 ± 2 °C, and primer dimers as well as non-specific amplification were minimized (verified using Oligo 7 software).

### Study design and participants

**Fig. 1:**
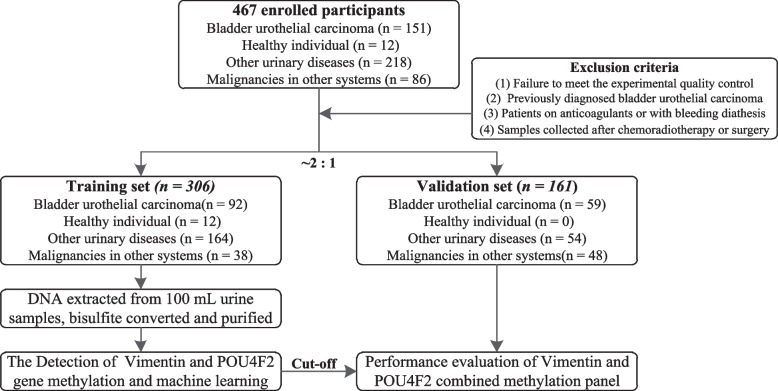
Study flow of participants in the study. A total of 467 urine samples were collected; methylation levels of Vimentin and POU4F2 were analyzed in 306 samples and subsequently validated in 161 samples using RT-PCR. All samples were obtained from the First Affiliated Hospital of Anhui Medical University, with ethical approval granted by its Medical Ethics Committee

We conducted a retrospective diagnostic study using urine samples, which were divided into training and validation sets for model development. Urine samples from 151 BC patients, 218 patients with other urinary diseases, 86 patients with malignancies in other systems, and 12 healthy individuals were collected from the First Affiliated Hospital of Anhui Medical University and split into training and validation sets at a 2:1 ratio. The training set (*n* = 306) consisted of 92 BC patients, 164 patients with other urinary diseases, 38 with malignancies in other systems, and 12 healthy individuals. The validation set (*n* = 161) included 59 BC patients, 54 patients with other urinary diseases, and 48 with malignancies in other systems (Fig. 1). Additional details of the subjects’ characteristics are provided in Table S1.

All participants underwent standard urological evaluations, including cystoscopy and computed tomography urography. BC diagnoses were made by attending physicians and confirmed through histopathological evaluation. The control group consisted of patients with other urinary diseases, without cystoscopically visible or histopathologically confirmed BC, and with no prior history of the disease. Meanwhile, urine samples from patients with malignancies in other systems, confirmed through histopathological or imaging examinations, were classified as the interference group. The interference group, experimental group, and control group samples were used not only for model training and fitting (for regression models) but also for post-model validation to assess the predictive performance and robustness of the established regression model.

### Sample processing and DNA extraction

Morning urine samples from cancer patients were collected prior to any surgical or therapeutic intervention. Approximately 100–200 mL of urine was collected and stored in collection tubes (Anhui Anlong Gene Technology Co., Ltd., Anhui, China). Freshly collected 100 mL urine samples were gently mixed, transferred to 50 mL centrifuge tubes, and centrifuged at 3500 rpm for 10 min using a benchtop centrifuge. The supernatant was discarded, and the cell pellets at the bottom were collected. One milliliter of PBS was added to the pellets, mixed, and centrifuged at 3500 rpm for 2 min. The supernatant was discarded, yielding purified urine cell pellets. DNA was extracted from the cell pellets using the Nucleic Acid Extraction and Purification Kit (Magnetic bead method) (Anhui Anlong Gene Technology Co., Ltd.). The purified DNA was transferred to a new tube and stored at −20 °C for subsequent methylation analysis. Urine cell sediments obtained by centrifugation can be stored at −70 °C ± 10 ℃ or lower for up to 12 months.

### DNA methylation analysis

The methylation status of two genes was analyzed by RT-PCR using ACTB as the reference gene [29]. RT-PCR was performed on the Applied Biosystems 7500 Fast Real-Time PCR System (Applied Biosystems, Foster City, CA, USA). DNA methylation analysis was carried out according to the manufacturer’s instructions provided with the diagnostic kit (Anhui Anlong Gene Technology Co., Ltd.). Each experiment included a positive control, a negative control, and a blank control without template DNA. The PCR amplification protocol was as follows: initial denaturation at 95 °C for 5 min, followed by 42 cycles of denaturation at 95 °C for 10 s and annealing at 58 °C for 30 s. The cycle threshold (Ct) value was recorded when the amplification curve of the FAM/ROX fluorescence signal exhibited an “S” shape or distinct exponential growth. Commercially available methylated human genomic DNA (Qiagen, Hilden, Germany) was used as the positive control. Water blanks and PCR mixtures were used as negative controls.

### Statistical analysis

Receiver operating characteristic (ROC) curves were used to evaluate the sensitivity, specificity, and accuracy of single or combined biomarkers for the classification of BC using IBM SPSS Statistics 24 (IBM, New York, NY). For single-gene performance analysis, the comparative Ct (ΔCt) method was used to calculate the Ct values of the target genes, based on the Ct values of the Vimentin and POU4F2 genes. A ΔCt_Vimentin_ or ΔCt_POU4F2_ ≤ 8.50 or 8.46 indicated positive methylation of Vimentin or POU4F2, respectively. For biomarker combination analysis, binary logistic regression analysis (Logitc−ABI7500= 4.047 − 0.286 × ΔCt_POU4F2_ − 0.235 × ΔCt_Vimentin_) was performed. The logistic regression formula was weighted with a *P*-value = 10/ [1 + exp(−Logitc_−ABI7500_)], ranging from 0 to 10. Based on ROC curve analysis, when the cutoff *P*-value was set at 4.392, the maximum Youden index (0.792) was observed. Therefore, the *P*-value cutoff was set as an integer value of 4.4.

Sensitivity was defined as the proportion of correctly identified positive BC samples among all BC samples.

Specificity was defined as the proportion of correctly identified negative samples among all normal/benign urinary disease and other malignancy samples. Positive predictive value (PPV; the probability that the disease is present given a positive test result) and negative predictive value (NPV; the probability that the disease is absent given a negative test result) were also calculated. The association between test positivity and demographic characteristics was assessed using the Chi-square test. Statistical significance was defined as *P* < 0.05. Odds ratios (OR) were calculated with 95% confidence intervals (CIs).

## Results

### ROC analysis of Vimentin and POU4F2 methylation biomarkers

**Fig. 2:**
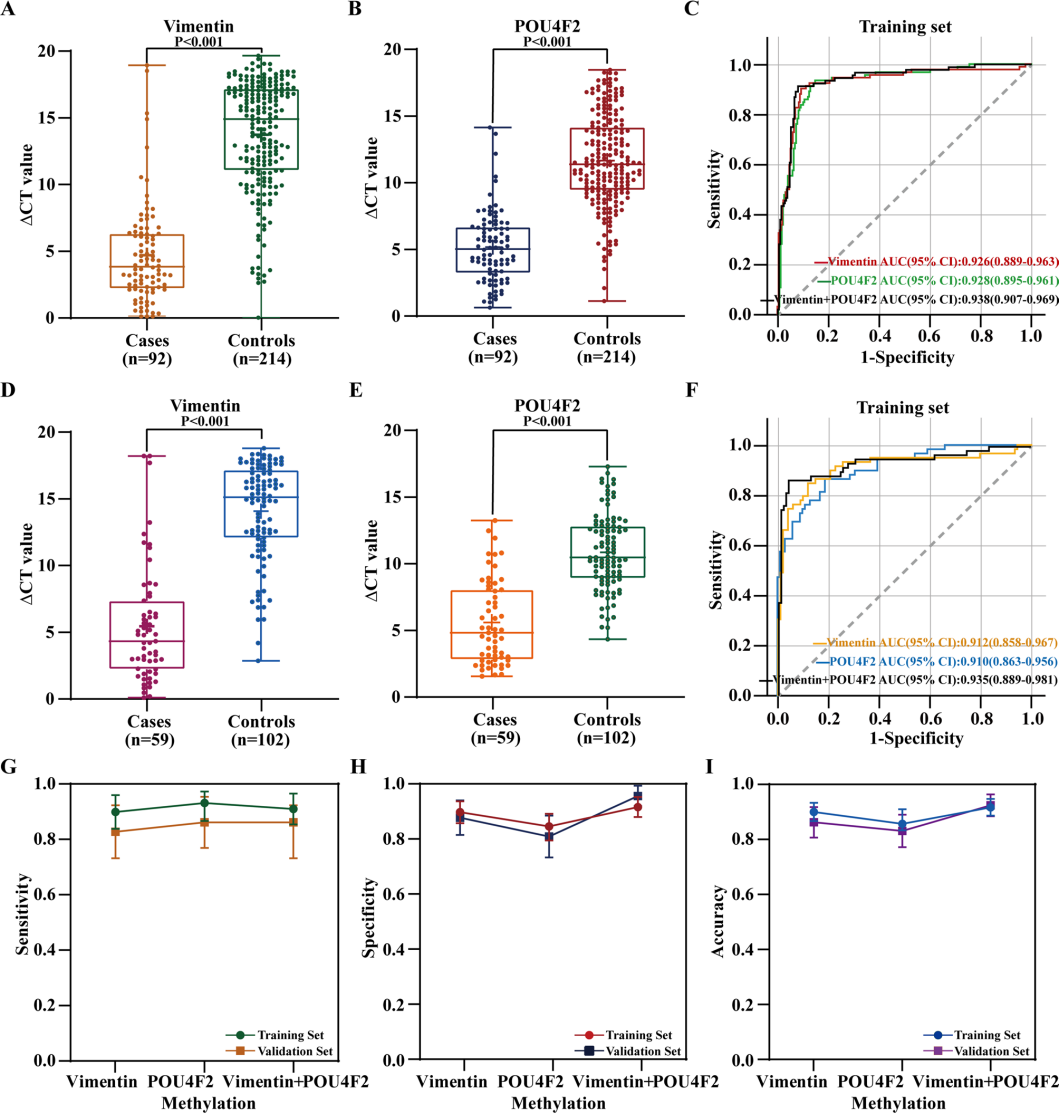
Methylation level distribution and diagnostic performance analysis of Vimentin and POU4F2, as well as the combined two-gene methylation panel, in BC cases and controls. **A**, **B** Methylation level distribution of Vimentin (**A**) and POU4F2 (**B**) in the training set. **C** ROC curves of Vimentin, POU4F2, and the combined Vimentin/POU4F2 methylation panel in the training set. **D**, **E** Methylation level distribution of Vimentin (**D**) and POU4F2 (**E**) in the validation set. **F** ROC curves of Vimentin, POU4F2, and the combined methylation panel in the validation set. **G**-**I** Bar plots depicting the sensitivities (**G**), specificities (**H**), and accuracies (**I**) for detecting BC using Vimentin, POU4F2, and the combined methylation panel in both the training and validation sets. Error bars represent 95% confidence intervals

The methylation levels of Vimentin and POU4F2 biomarkers in urine cell pellets from BC cases, controls, and interference groups were analyzed using RT-PCR. Results showed that mean ∆CT values of Vimentin (4.61) and POU4F2 (5.20) were significantly lower in cases compared to controls (13.73 and 11.66, respectively) (Fig. 2A and B) in the training set. The average ∆CT values of Vimentin (5.34) and POU4F2 (5.30) were also significantly lower in cases compared to controls (14.10 and 10.91, respectively) (Fig. 2D-E) in the validation set. These results indicate that Vimentin or POU4F2 methylation can be reliably measured.

ROC analysis demonstrated the diagnostic efficacy of Vimentin and POU4F2 methylation in urine DNA, with AUCs of 0.926 (95% CI: 0.889–0.963) and 0.928 (95% CI: 0.895–0.961), respectively, for identifying BC (Fig. 2C). The sensitivity, specificity and accuracy of Vimentin were 90.22% (95% CI: 0.841–0.963), 90.19% (95% CI: 0.862–0.942) and 90.10% (95% CI: 0.868–0.934), respectively. POU4F2 showed a sensitivity of 93.48% (95% CI: 0.870–0.977), specificity of 85.05% (95% CI: 0.800–0.897) and accuracy of 87.58% (95% CI: 0.835–0.912) (Fig. 2G-I). While in cases where sensitivity (91.30%, 95% CI: 0.857–0.969) is comparable to that of the single-gene panel, the Vimentin/POU4F2 combined methylation panel yielded a high diagnostic specificity of 92.06% (95% CI: 0.884–0.957) and accuracy of 91.83% (95% CI: 0.888–0.949) (Fig. 2G-I), with an AUC of 0.938 (95% CI: 0.907–0.969) in the training set (Fig. 2C).

Additionally, the diagnostic performance of the validation set was marginally lower compared to that of the training set. The sensitivity, specificity and accuracy of Vimentin were 83.05% (95% CI: 0.735–0.926), 88.24% (95% CI: 0.820–0.945) and 86.34% (95% CI: 0.808–0.919), with an AUC of 0.912 (95% CI: 0.858–0.967) (Fig. 2F-I); POU4F2 demonstrated a sensitivity of 86.44% (95% CI: 0.772–0.957), specificity of 81.37% (95% CI: 0.738–0.889) and accuracy of 83.23% (95% CI: 0.773–0.891), with an AUC of 0.910 (95% CI: 0.863–0.956) (Fig. 2F-I). The Vimentin/POU4F2 combined methylation biomarkers achieved an AUC of 0.935 (95% CI: 0.889–0.981), with sensitivity, specificity and accuracy of 86.44% (95% CI: 0.772–0.957), 96.08% (95% CI: 0.923–0.998) and 92.55% (95% CI: 0.886–0.965) (Fig. 2F-I). These results indicate that the combined methylation assessment has higher specificity (low false positive rate) and accuracy compared to single-gene methylation biomarkers assessment.

### Diagnostic value of Vimentin/POU4F2 combined methylation biomarkers in urine DNA

**Fig. 3:**
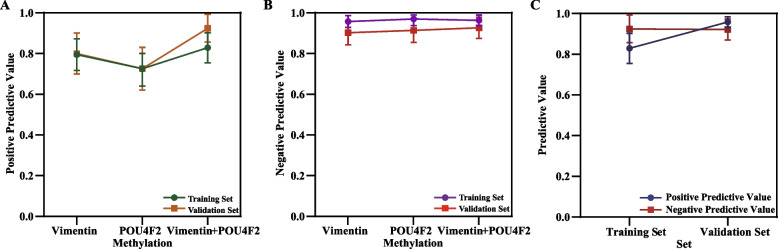
Distributions of PPV and NPV for Vimentin, POU4F2, and the combined Vimentin/POU4F2 methylation panel in the training and validation sets. **A**, **B** PPV (**A**) and NPV (**B**) distributions of Vimentin, POU4F2, and the combined methylation panel in the training set. **C** Comparison of PPV and NPV for the combined methylation panel between the training and validation sets. Error bars represent 95% confidence intervals

Currently, the main screening methods for BC have notable limitations, including overdiagnosis, low sensitivity, and high false-positive rates. These limitations hinder effective early-stage detection, reducing the reliability and utility of early screening. Given the high false-positive rates and overdiagnosis associated with imaging-based screening, unnecessary invasive procedures should be minimized, particularly in patients with benign urinary conditions. The PPV and NPV of Vimentin were 80.33% (95% CI: 0.703–0.904) and 90.00% (95% CI: 0.841–0.959), respectively. For POU4F2, the PPV and NPV were 72.86% (95% CI: 0.624–0.834) and 91.21% (95% CI: 0.853–0.971), respectively (Fig. 3A and B) in the validation set. The combined Vimentin/POU4F2 methylation panel achieved a PPV of 92.73% (95% CI: 0.859–0.995) and an NPV of 92.45% (95% CI: 0.873–0.976) (Fig. 3C). These results indicate that the two-gene methylation panel achieved significantly higher PPV and NPV in both the training and validation sets. This improvement enhanced diagnostic efficiency and yielded better alignment with clinical diagnostic outcomes compared to single-gene assessments.

### Diagnostic performance of the dual-gene methylation panel in different subtypes

**Fig. 4:**
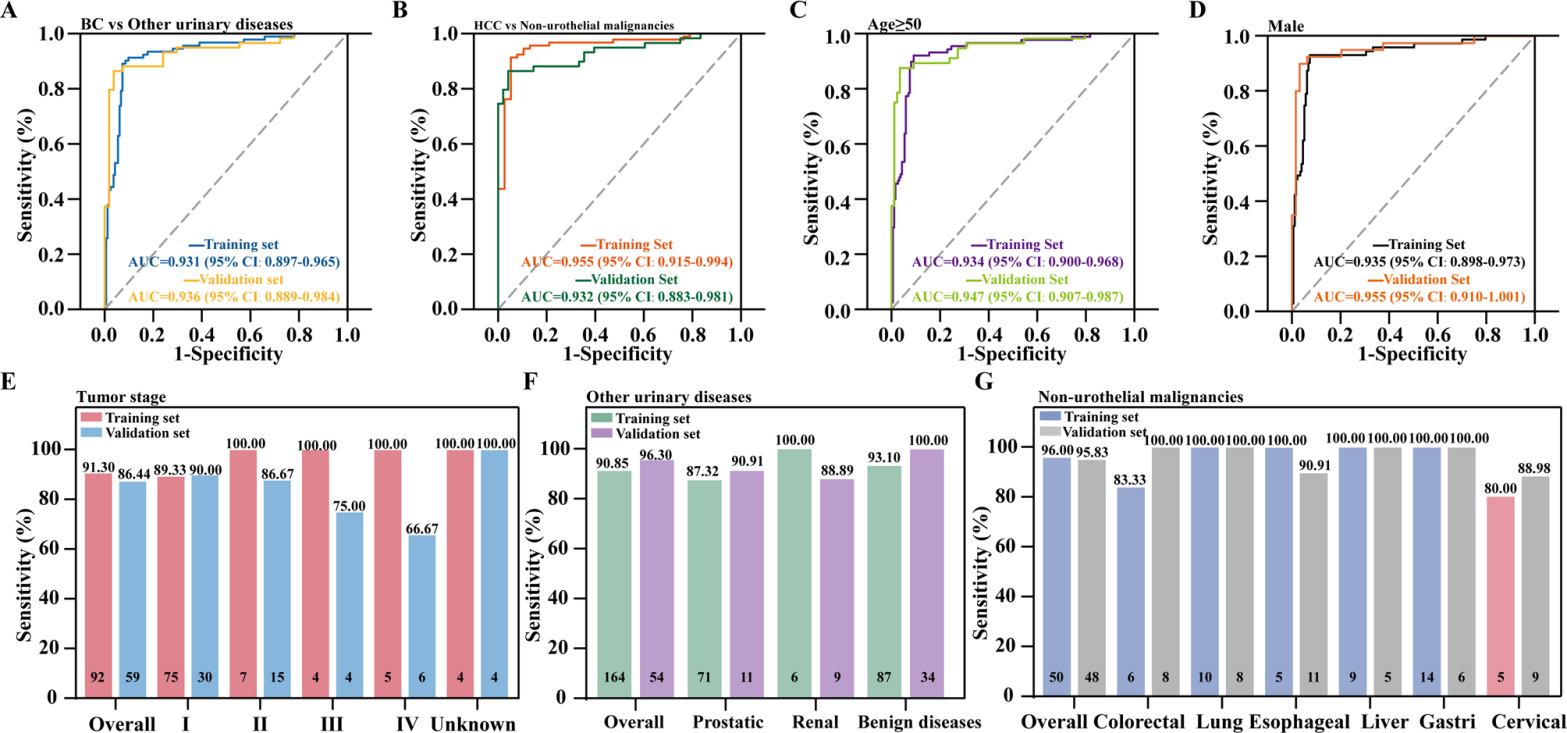
Diagnostic performance of the dual-gene methylation panel across different subtypes. **A** ROC curve plots of the dual-gene methylation panel for distinguishing BC patients from individuals with other urinary diseases in the training and validation sets, including those with prostate cancer, renal cancer, and benign urinary diseases. **B** ROC curve plots of the dual-gene methylation panel for distinguishing BC patients from individuals with non-urothelial malignancies in the training and validation sets, including those with colorectal, lung, esophageal, liver, gastric, and cervical cancer. **C** ROC curve plots of the dual-gene methylation panel for identifying BC and non-BC patients aged ≥ 50 in the training and validation sets. **D** ROC curve plots of the dual-gene methylation panel for identifying male BC and non-BC patients in the training and validation sets. **E** Sensitivity of the dual-gene methylation panel in BC patients at specified stages in the training and validation sets. Tumors of TNM stage were stratified as stages I, II, III, and IV. **F**, **G** Specificity of the dual-gene methylation panel for the indicated types of other urinary diseases (**F**) and non-urothelial malignancies (**G**) in the training and validation sets

A comprehensive analysis was conducted to evaluate potential biases and diagnostic efficacy in demographic and clinicopathological characteristics, including age, gender, and tumor stage, of the Vimentin/POU4F2 combined methylation panel in urine DNA. Detailed data results are provided in Table S2. ROC analysis demonstrated that, when differentiating BC from patients with other urinary diseases, the AUCs of the two-gene methylation panel in the validation set were 0.936 (95% CI: 0.889–0.984) (Fig. 4A). The sensitivity was 86.44% (95% CI: 0.772–0.957) and the specificity was 96.30% (95% CI: 0.913–0.993) (Fig. 4F). Specifically, in patients with prostate cancer, the specificity was 90.91%; in renal cancer patients, it was 88.89%; and in patients with benign urinary diseases, it was 100% in the validation set (Fig. 4F, Tab. S2). Additionally, when differentiating BC from patients with non-urothelial malignancies, the AUCs of the Vimentin/POU4F2 methylation panel in the validation set were 0.932 (95% CI: 0.883–0.981) (Fig. 4B). The sensitivity was 86.44% (95% CI: 0.772–0.957) and the specificity was 95.83% (95% CI: 0.902–1.000) (Fig. 4G). Specifically, for patients with colorectal, lung, esophageal, liver, gastric, and cervical cancer, the specificities were 100.00%, 100.00%, 90.91%, 100.00%, 100.00% and 88.89%, respectively, in the validation set (Fig. 4G, Tab. S2).

In the age and gender analysis, the AUCs of the Vimentin/POU4F2 methylation panel for BC patients aged ≥ 50 years in the validation set were 0.947 (95% CI: 0.907–0.987) (Fig. 4C), with sensitivity and specificity of 86.96% and 96.59%, respectively (Tab. S2). In males from the validation set, the AUC of the dual-gene panel was 0.955 (95% CI: 0.910–1.001) (Fig. 4D), with sensitivity of 86.96% and specificity of 95.77% (Tab. S2). Stratification by age and gender did not significantly affect the panel’s sensitivity or specificity in either the training or validation sets (Tab. S2). Across BC tumor stages, the dual-gene methylation panel demonstrated high sensitivity for early stages of BC, with sensitivity of 90.00% and 86.67% for stage I and stage II, respectively, in the validation set (Fig. 4E). Additionally, in the analysis of different grades of BC, the Vimentin/POU4F2 methylation panel demonstrated high sensitivity regardless of BC grade. Overall, the ROC analysis, diagnostic value, and performance demonstrated that the Vimentin/POU4F2 methylation panel is a highly effective and noninvasive method for detecting BC. It offers significant sensitivity and specificity for distinguishing BC from other urinary diseases, and demonstrates promising clinical diagnostic value. However, due to the limited sample size, more independent clinical test samples are needed to further demonstrate the diagnostic performance of the Vimentin/POU4F2 methylation panel in future clinical trials.

## Discussion

BC is one of the most prevalent malignancies of the urinary system, with both morbidity and mortality rates consistently increasing each year [30]. At present, cystourethroscopy, urinary cytology, and computed tomography urography remain the gold standards for diagnosing BC [3]. However, cystourethroscopy is costly and invasive, leading to significant patient discomfort, with sensitivity varying based on several factors [31]. Conventional urine cytology for non-invasive BC detection and disease monitoring is hindered by low sensitivity, particularly for low-grade carcinoma [27]. Therefore, there is an urgent need to develop reliable, non-invasive methods to accelerate BC diagnosis, thereby enabling early detection to improve the five-year survival rate and reduce the disease burden.

Reliable clinical biomarkers for tumors should, ideally, be non-invasive, demonstrating high specificity and sensitivity, thus offering clinicians a useful tool to overcome the limitations of cystourethroscopy and imaging, and to prioritize patients for invasive diagnostic interventions [28]. Specific markers, such as NMP-22 and BTA, which are approved by the US Food and Drug Administration (FDA), exhibit higher sensitivity, particularly for low-grade tumors, although their specificity is lower than that of urine cytology [32]. DNA methylation is a prevalent epigenetic modification that directly influences the binding of DNA-binding proteins to their respective sites, thereby regulating the expression of associated genes [33]. Studies have shown that aberrant DNA methylation in tumor-specific genes is associated with histological stage, grade, recurrence, and progression of urological tumors, including prostate, bladder, renal, and testicular cancers. It can be detected early, prior to clinical diagnosis [34]. Therefore, by detecting tumor cells shed in urine, DNA methylation could serve as a non-invasive approach for detecting and monitoring BC.

Previous studies have indicated that most patients are hesitant to replace cystoscopy with urinary tests that have sensitivities lower than 90%. In this retrospective biomarker study, we developed a novel methylation marker panel comprising Vimentin/POU4F2 for detecting BC, achieving an AUC of 0.935, with sensitivity, specificity, and accuracy of 86.44%, 96.08% and 92.55% for diagnosing BC (Fig. 2). Notably, for patients with stage I or low-grade BC, the sensitivity was 90.00% for both (Fig. 4 and Tab. S2). Additionally, for patients with other urinary diseases, the Vimentin/POU4F2 combined methylation panel exhibited high specificity (96.30%), particularly for benign urinary diseases, where the specificity reached 100.00% (Fig. 4 and Tab. S2). Furthermore, this study found that in the training set, the sensitivity of the combined methylation detection panel (91.30%) was slightly lower than that of the POU4F2 marker alone (93.48%), with an absolute difference of only 2.18%. However, its specificity and overall accuracy were significantly improved; for example, specificity increased from 85.05% to 92.06% and accuracy rose from 87.58% to 91.83% (Fig. 2). This phenomenon reflects the classic “sensitivity–specificity trade-off” in the development of diagnostic markers. Although the sensitivity of the combined methylation detection panel slightly decreased, its specificity increased more significantly (with an absolute difference of 7.01%), and the overall accuracy improved substantially, indicating a notable reduction in the overall risk of misdiagnosis (false negatives + false positives). Key subgroup analysis revealed that the combined detection maintained high sensitivity for early-stage tumors and high-grade cancers, without significantly compromising early detection capability. In bladder cancer screening, high specificity is as crucial as sensitivity in reducing overtreatment, particularly for healthy individuals or patients with benign urological diseases (such as inflammation and stones). Moreover, in the analysis of the diagnostic value of the combined Vimentin/POU4F2 methylation biomarkers in urine DNA, the combined Vimentin/POU4F2 methylation detection achieved a positive predictive value of 92.73% (95% confidence interval: 0.859–0.995) and a negative predictive value of 92.45% (95% confidence interval: 0.873–0.976) (Fig. 3C). These results suggest that this dual-gene methylation detection combination has a low rate of missed diagnoses and false positives, and its consistency with clinical diagnostic results is superior to that of single-gene assessments.

Interestingly, our study identified a significant correlation between the methylation status of Vimentin/POU4F2 and physiological age. The positivity rate for Vimentin/POU4F2 combined methylation was higher in patients aged 50 years or older. It is well established that age significantly influences DNA methylation. Numerous studies have shown that aging is associated with changes in tumor suppressor genes and global genome hypomethylation [35, 36]. However, the precise mechanisms by which age induces hypermethylation of Vimentin/POU4F2 remain unclear. This could be related to increased expression of DNMTs and enhanced DNA methylation in these regions, or to an increase in detected tumor cells in urine samples with aging [37]. In summary, our retrospective study demonstrates that the Vimentin and POU4F2 combined methylation biomarker panel is a novel, cost-effective, and non-invasive method that offers significant sensitivity and specificity for distinguishing BC from other urinary diseases, showing potential clinical diagnostic value, particularly for patients with stage I and low-grade BC. Additionally, urine samples can be collected at home and sent to any laboratory. This makes the method easily applicable and truly non-invasive for routine clinical care.

### Limitations and strengths of the study

Nevertheless, several limitations of this study warrant consideration. First, the applicability of the Vimentin/POU4F2 methylation panel to other urothelial carcinomas—such as ureteral, renal pelvic, and upper tract tumors—remains to be determined. Future studies should include urine samples from a broader range of urothelial carcinoma patients to further evaluate the panel’s diagnostic performance. Second, this study employed a case–control design. To enhance clinical relevance and validate our findings, a large multicenter trial is warranted. A head-to-head comparison between the Vimentin/POU4F2 panel and urine cytology in matched samples is essential to fully assess their relative clinical utility. However, due to the study’s focus on biomarker discovery, model construction, and internal validation, cytology tests were not conducted concurrently on the same sample sets owing to design and resource constraints. A rigorous head-to-head comparison with urine cytology will be performed in a large, independent external validation cohort. Key strengths of this study include the strong diagnostic performance of novel urinary methylation markers and the practicality of home-based sample collection with remote laboratory submission. This renders the approach widely applicable and truly non-invasive for routine clinical use. Additionally, the method requires only two methylation markers and one reference gene in a simplified, cost-effective PCR assay, enabling rapid deployment in routine diagnostic laboratories.

## Conclusion

In conclusion, the Vimentin/POU4F2 methylation biomarkers exhibit high specificity, sensitivity, and diagnostic value for non-invasive BC detection, supporting their cost-effective clinical applicability. Collectively, these findings indicate that urinary gDNA methylation biomarkers offer a reliable, non-invasive, and cost-effective adjunct for future BC diagnosis.

## Supplementary Information

Supplementary Material 1.

## Data Availability

The datasets used and analyzed during the current study are available from the corresponding author on reasonable request. Subjects’ data are not publicly available as they contain information that could compromise participants’ consent and confidentiality.

## Abbreviations

BC: Bladder carcinoma
POU4F2: POU class 4 homeobox 2 gene
RT-PCR: Real-Time PCR
ROC: Receiver operator characteristic
PPV: Positive predictive value
NPV: Negative predictive value
AUC: Area under the curve
CI: Confidence interval
