# Supplementary material for: The diagnostic performance of a noninvasive urine-based methylation biomarkers Vimentin/POU4F2 to detect bladder carcinoma
Source: BMC Cancer. 2025 Sep 30;25:1460. doi: 10.1186/s12885-025-14795-5 (PMC12482282; doi:10.1186/s12885-025-14795-5)
Supplement: Supplementary file 1 — Supplementary Material 1. [file 12885_2025_14795_MOESM1_ESM.docx]

CATCAAGCAGATAAAGGGGGCTGTCAAAATGTGCCTTTAAGCGGTGATTATGCAGAAATACGCACCGGTGGGGTTGGAAGACAGGCTCTCCGCATAGCCTCCGAGCCCCCGCCGCCGCTGCTGCCACTGCTGCTGGAGCTGCTGCTTCGGCCTCCGCCGCCGCCGCCGCCGCCGCCGCCGCCGCCGCCACCACCAGCGTTGCTCGAGCTGCTGGGGGAGCTGGCCGAGGGCGCGATGGGAGCCGAGGAGCCCGGCGAGGTGCTGTGCAGTGCCGAGTACTTGGGCTCCACGTGCAGGCTGCCGCCGTGCGGCATGCTAAACGCCTGCTTGCTGTTCAGGGACATCATCATCATCTTCCCGCCCGCCGGGGCGCTGGTCCGTAGAGGCACTCACTGGTCCCGGCTGCCGGGACCCTCCGAGAAGTGCCGGGCCAGCCGGGGCTGGAGCTGGGAAGGGCTGTGCGAAGTTGAGCTCACCCGCCGCCGCCTCCGGACTCTGTACGCCTGATCTCGGCTACGCGCTCCTTCGCCGCCTCTGCTGTCAGTCTGAACGGATGCCTGACTCCGCTTGCCGCTAGCTGCAGACCTCGGCACCCGAAACTGCCCCCGCTCCTCGCGGCCGGCGAGTTATACTCCCCTCTCCCCGCGCCTACCCCCGCGCCCCCCGCCCTCCCTCTCTCCCTCTCTCCCGCGCGCCTCCGCCCTCCCTTGCCAGGAAACGCCGGCTCCTGTTCGGTTTCTCGCGTTGCCCCTTCTTCCTCTCTACTCCCCTCAGGCTTGAGTCCTTTCTGCTCTCTCAGCACTCGCCTCGCGCTCCCCCACCGCGCCCTCCGCCGCCTCTTTTCTGCCCCCGCCCGTGCTCTGTTGCCGCCGCCCCTTCGCAGCGTCCCGCGGCTGCTACTCACAATCGCGCCCCCACTCAACCCCACCCAAATGGCCAGGTCCGCGCTCCAGTCGCCGGGCAGTGCCTACCTAAGGACCAGCCTCCAGCCCCTCCCGCTTCTTCTGCCGGGTCTCTGTC

**Figure S1. The genome version for the probe coordinates of POU4F2 (Chr4: -147559626 — -147560645).** The sequences of “GAGCTCACCCGCCGCCGCCTCC” and “CGCCTCTGCTGTCAGTCTGAAC” are the upstream and downstream primers, respectively; the sequence of “CTGTACGCCTGATCTCGGCTACGCG” is the probe.

CCCGCTCCTTTGCCCGCGGGTCTCCCCGCCTGACCGCAGCCCCGAGACCGCCGCGCACCTCCTCCCACGCCCCTTTGGCGTGGTGCCACCGGACCCCTCTGGTTCAGTCCCAGGCGGACCCCCCCCTCACCGCGCGACCCCGCCTTTTTCAGCACCCCAGGGTGAGCCCAGCTCAGACTATCATCCGGAAAGCCCCCAAAAGTCCCAGCCCAGCGCTGAAGTAACGGGACCATGCCCAGTCCCAGGCCCCGGAGCAGGAAGGCTCGAGGGCGCCCCCACCCCACCCGCCCACCCTCCCCGCTTCTCGCTAGGTCCCTATTGGCTGGCGCGCTCCGCGGCTGGGATGGCAGTGGGAGGGGACCCTCTTTCCTAACGGGGTTATAAAAACAGCGCCCTCGGCGGGGTCCAGTCCTCTGCCACTCTCGCTCCGAGGTCCCCGCGCCAGAGACGCAGCCGCGCTCCCACCACCCACACCCACCGCGCCCTCGTTCGCCTCTTCTCCGGGAGCCAGTCCGCGCCACCGCCGCCGCCCAGGCCATCGCCACCCTCCGCAGCCATGTCCACCAGGTCCGTGTCCTCGTCCTCCTACCGCAGGATGTTCGGCGGCCCGGGCACCGCGAGCCGGCCGAGCTCCAGCCGGAGCTACGTGACTACGTCCACCCGCACCTACAGCCTGGGCAGCGCGCTGCGCCCCAGCACCAGCCGCAGCCTCTACGCCTCGTCCCCGGGCGGCGTGTATGCCACGCGCTCCTCTGCCGTGCGCCTGCGGAGCAGCGTGCCCGGGGTGCGGCTCCTGCAGGACTCGGTGGACTTCTCGCTGGCCGACGCCATCAACACCGAGTTCAAGAACACCCGCACCAACGAGAAGGTGGAGCTGCAGGAGCTGAATGACCGCTTCGCCAACTACATCGACAAGGTGCGCTTCCTGGAGCAGCAGAATAAGATCCTGCTGGCCGAGCTCGAGCAGCTCAAGGGCCAAGGCAAGTCGCGCCTGGGGGACCTCTACGAGGAGGAGATGCGGGAGCTGCGCCGGCAGGTGGACCAGCTAACCAACGACAAAGCCCGCGTCGAGGTGGAGCGCGACAACCTGGCCGAGGACATCATGCGCCTCCGGGAGAAGTAAGGCTGCGCCCATGCAAGTAGCTGGGCCTCGGGAGGGGGCTGGAGGGAGAGGGGAACGCCCCCCCGGCCCCCGCGAGAGCTGCCACGCCCTTGGGGATGTGGCCGGGGGGAGGCCTGCCAGGGAGACAGCGGAGAGCGGGGCTGTGGCTGTGGTGG

**Figure S2. The genome version for the probe coordinates of VIMENTIN (Chr10: +17270244 — +17272636).** The sequences of “GTCCACCAGGTCCGTGTCCTCGTC” and “CGAGCCGGCCGAGCTCCAGCC” are the upstream and downstream primers, respectively; the sequence of “CGCAGGATGTTCGGCGGCCCGGGCA” is the probe.

**Table S1. The demographic and clinicopathologic characteristics of study participants**

| **Characteristics** | **Training set** | | | **Validation set** | | |
| --- | --- | --- | --- | --- | --- | --- |
| **Clinical** | **Case (*n* = 92)** | **Control (*n* = 214)** | ***P*** | **Case (*n* = 59)** | **Control (*n* = 102)** | ***P*** |
| **Age (Years)** |  |  |  |  |  |  |
| Median Age | 70 | 67 | =0.002 | 70 | 65 | =0.037 |
| Mean age | 69.15 | 63.94 |  | 66.88 | 64.55 |  |
| Age Range | 37-95 | 21-98 |  | 34-89 | 21-96 |  |
| **Gender (*n, %*)** |  |  |  |  |  |  |
| Male | 71 (77.17) | 177 (82.71) | <0.001 | 46 (77.97) | 71 (69.61) | <0.001 |
| Female | 21 (22.83) | 37 (17.29) |  | 13 (22.03) | 31 (30.39) |  |
| **Grade (*n, %*)** |  |  |  |  |  |  |
| Low | 35 (38.04) |  |  | 20 (33.90) |  |  |
| High | 48 (52.18) |  |  | 35 (59.32) |  |  |
| Unknown | 9 (9.78) |  |  | 4 (6.78) |  |  |
| **Stage** **(*n, %*)** |  |  |  |  |  |  |
| I | 75 (64.66) |  |  | 30 (46.15) |  |  |
| II | 7 (6.03) |  |  | 15 (23.08) |  |  |
| III | 4 (3.45) |  |  | 4 (6.15) |  |  |
| IV | 2 (1.72) |  |  | 6 (9.23) |  |  |
| Unknown | 4 (3.45) |  |  | 4 (6.15) |  |  |
| **Histopathology (*n, %*)** |  |  |  |  |  |  |
| Bladder urothelial carcinoma | 92 (79.31) |  |  | 59 (90.76) |  |  |
| Urinary system diseases |  |  |  |  |  |  |
| Prostatic carcinoma |  | 71 (43.29) |  |  | 11 (20.37) |  |
| Renal carcinoma |  | 6 (3.66) |  |  | 9 (16.67) |  |
| Benign urinary diseases |  | 87 (50.05) |  |  | 34 (62.96) |  |
| Other Malignancies |  |  |  |  |  |  |
| Colorectal cancer |  | 6 (15.79) |  |  | 8 (16.67) |  |
| Lung cancer |  | 10 (26.32) |  |  | 8 (16.67) |  |
| Esophageal cancer |  | 5(13.16) |  |  | 11 (22.92) |  |
| Liver cancer |  | 2 (5.25) |  |  | 4 (8.33) |  |
| Gastric cancer |  | 10 (26.32) |  |  | 8 (16.67) |  |
| Cervical cancer |  | 5 (13.16) |  |  | 9 (18.75) |  |
| Healthy individual |  | 12 |  |  |  |  |

**Table S2 Diagnostic performance of the Vimentin/POU4F2 combined methylation panel in different subtypes**

| **Types of Cancer** | **Training set (Vimentin+POU4F2)** | | **Validation set (Vimentin+POU4F2)** | |
| --- | --- | --- | --- | --- |
|  | Sensitivity | Specificity | Sensitivity | Specificity |
| **Age (*%, n*)** |  |  |  |  |
| ≥ 50 | 92.05 (81/88) | 90.96 (171/188) | 85.96 (49/57) | 96.59 (85/88) |
| < 50 | 75.00 (3/4) | 100.00 (26/26) | 100.00 (2/2) | 92.86 (13/14) |
| **Gender (*%, n*)** |  |  |  |  |
| Male | 92.96 (66/71) | 92.66 (164/177) | 86.96 (40/46) | 95.77 (68/71) |
| Female | 85.71 (18/21) | 89.19 (33/37) | 84.62 (11/13) | 96.77 (30/31) |
| **Grade (*n, %*)** |  |  |  |  |
| Low | 82.86 (29/35) |  | 90.00 (18/20) |  |
| High | 95.83 (46/48) |  | 82.86 (29/35) |  |
| Unknown | 100.00 (9/9) |  | 100.00 (4/4) |  |
| **Stage (*%, n*)** |  |  |  |  |
| I | 89.33 (67/75) |  | 90.00 (27/30) |  |
| II | 100.00 (7/7) |  | 86.67 (13/15) |  |
| III | 100.00 (4/4) |  | 75.00 (3/4) |  |
| IV | 100.00 (2/2) |  | 66.67 (4/6) |  |
| Unknown | 100.00 (4/4) |  | 100.00 (4/4) |  |
| **Histopathology (*n, %*)** |  |  |  |  |
| Bladder urothelial carcinoma | 91.30 (84/92) |  | 86.44 (51/59) |  |
| Other urinary diseases |  |  |  |  |
| Prostatic cancer |  | 87.32 (62/71) |  | 90.91 (10/11) |
| Renal cancer |  | 100.00 (6/6) |  | 88.89 (8/9) |
| Benign urinary diseases |  | 93.10 (81/87) |  | 100.00 (34/34) |
| Other Malignancies |  |  |  |  |
| Colorectal cancer |  | 83.33 (5/6) |  | 100.00 (8/8) |
| Lung cancer |  | 100.00 (10/10) |  | 100.00 (8/8) |
| Esophageal cancer |  | 100.00 (5/5) |  | 90.91 (10/11) |
| Liver cancer |  | 100.00 (2/2) |  | 100.00 (4/4) |
| Gastric cancer |  | 100.00 (10/10) |  | 100.00 (8/8) |
| Cervical cancer |  | 80.00 (4/5) |  | 88.89 (8/9) |
| Healthy individuals |  | 100.00 (12/12) |  |  |
